# Indents

**Published:** 1919-09

**Authors:** 


### INDENTS

**☞Brief Articles of Technical or Scientific Value will receive notice and comment. Contributions invited.**

---

MOBILIZE FOR PEACE. The armistice has been signed, but the Red Cross is waging a war that knows no armistice—a war against disease, unhealthy living conditions, starvation—all the lingering poisons of the past.

The Red Cross asks you to hold fast to the ranks; to carry on in the work of restoration; to keep the faith with the widows and orphans of the heroic millions who died on Europe's Calvary that you might live. Are you going to sign an armistice before *this* war is over?

The Red Cross has never been content with temporary makeshift methods of relief—the work must be well done or not at all, and this work of war relief has only just begun. World-wide distress has followed in the wake of this war. The Red Cross needs workers as never before. It needs *your* heart, *your* brain, your *hands*—could you devote them to a better cause?

The third Red Cross roll call will be held November first to eleventh. The goal is twenty million members. There is need for the immediate services of one million volunteer workers to enroll them.

Are you ready to answer the call?

---

LOCAL ANESTHESIA. The objections to the local anesthesia are both real and imaginary. There is undoubtedly a class of cases in which general anesthesia should always be used. There is, I believe, also a class in which local anesthesia should always be used. That the latter class could be greatly increased with an improvement over the results obtained at present in the large clinics I also firmly believe. The objection that wide explorations can not be made under local anesthesia is to some extent true, and yet it is to a large degree offset by the fact that wide explorations not made under the direction of the eye seldom give authentic information.

Working under local anesthesia requires more time, but experience and proper equipment bring this much discussed factor into comparative insignificance. The strain on the surgeon is undoubtedly greater when working under local anesthesia; but if we accept the dictum that the all-important factor is the patient's welfare, this objection must be eliminated, and the experience of the surgeon will here, again, make a decided difference. During recent years I find much more comfort in operating on patients under local than under general anesthesia.

The use of local anesthesia should no longer be confined to minor surgery and to cases in which the patient apparently can not undergo the ordeal of taking a general anesthetic.

With proper equipment and technic, a large proportion of major surgery may be done under local anesthesia with greater safety, less trauma, more completeness and less post-operative discomfort than when general anesthesia is employed.

Children lend themselves readily to the method.—Dr. R. E. Farr, in *Journal A. M. A.*

---

THE AMERICAN CREED. It's a pretty good creed that turned up in a prize contest in Baltimore—worth all of the $1,000 that the city paid for it. It is called "The American's Creed," and it reads:

"I believe in the United States of America as a government of the people, by the people, for the people; whose just powers are derived from the consent of the governed; a democracy in a republic; a sovereign nation of many sovereign states; a perfect union, one and inseparable, established upon those principles of freedom, equality, justice and humanity for which American patriots sacrificed their lives and fortunes.

"I therefore believe it is my duty to my country to love it, to support its constitution, to obey its laws, to respect its flag and to defend it against all enemies."

It's a good test of Americanism. It can readily be applied to our own acts and words, and those of other people. By it any public act or utterance can be judged. If any man says he is an American, and behaves inconsistently with this creed, he is lying.

---

An Age of Quackery. A correspondent having called our attention to a freakish article appearing in a magazine of the "health fad" or "physical culture" type, it became necessary to buy a copy of this magazine. So much by way of apology. In clipping the various advertisements from this magazine for the propaganda files, we discovered in the fifty-seven varieties of fakes, medical, quasi-medical, and otherwise, the dominant note of the magazine's advertisers: short cuts! Short cuts to life, liberty and the pursuit of happiness. Short cuts to health, of course, predominate. One learns that he may cure himself of almost anything from soft corns to cirrhosis of the liver by means of the various "internal baths" so plausibly presented by numerous gentlemen who wish to relieve humanity—of surplus cash. You can be made "a one hundred per cent. man" by at least six different methods of physical training, each of which is unique and entirely different from its five worthless competitors. Are your eyes weak? There is a short cut cure for them! Are you deaf? There is a short cut to perfect hearing! Are you ruptured? There is a short cut hernia cure. Do you crave large busts? Presto change, you may have them. Every faddist shrewd enough to keep out of the insane asylum seems to be exploiting his particular panacea. One thing must be said for the advertising department of this magazine: it is at least consistent. It does not confine the short cut methods to the relief and cure of human ailments. Anyone with a short cut—and the wherewithal to pay for exploiting it—is welcome. One is able, so the advertisers assure us, to "learn shorthand in seven days;" to "increase your will power in one hour;" to "gain a thorough knowledge of law in your spare time;" to learn in one evening "the secret of being a convincing talker;" to learn to play the piano "in quarter the usual time at quarter the usual cost, and all by correspondence." Verily, we live in an age of quackery. And all the quacks are not in the medical profession, nor are all the nostrums in the "patent medicine" field. Economists tell us that never in the history of the country has there been so much money per capita as today. As a result, there are abroad in the land gentlemen who, figuratively speaking, wish you to invest. After looking through this magazine, it became obvious that the get-rich-quick fraternity does not confine its talents to selling oil stocks, real estate, or "mountain canaries."—*Journal A. M. A.*, May 17.

---

THE COUNTRY AND THE CITY DENTIST. Most country dentists own their own homes, and drive their own automobiles, usually of good make; yet they foolishly envy their city brother because they hear that he gets ten dollars an hour, uses the X-ray and gets two dollars and upward for root canal treatment.

I met such a country dentist once up in New Hampshire, and this was his tale of woe. He asked me what the law was in New York state, and whether I thought he could get a license to practice there. His reason for wishing to invade the big city was that his practice had reached six thousand per year and had not increased for five years. I asked him a few questions and learned that he had the "best office in town" and paid $20 a month rent. He owned his own house; had a fine pair of horses, and likewise an automobile, and had saved $3,000 annually for five years. Yet he wished to go to New York and asked my advice. Let me repeat what I told him:

"Go to New York, and with your skill, I am sure you will make a success, but it will take time. Ten years from now you will have a practice worth ten or twelve thousand dollars a year. You will have spent all the fifteen thousand that you now have; you will have office and living expenses amounting to from eight to fourteen thousand dollars a year, depending upon whether you remain a country man and a saver, or, what is more likely, grow to be a city man and a spender. You will live half of these ten years in a flat and after that will be a cliff dweller in an apartment. But if you want to live in a house such as you have now, with windows on four sides and 'green grass growing all around,' the rent will be about ten thousand per annum. But then, of course, you will be getting ten dollars an hour, whereas now you only earn three."

Strange to say, that man took my advice and remained in his country town and kept pegging along with his country practice. They tell me he is worth over a hundred thousand now, but perhaps he did not make it all filling teeth. He has a boy too, at Harvard, who is some pumpkins. Quite a city chap, and not only drives a bright yellow racing car, but eats his peas with a fork.—Dr. Ottolengui, in *Around the Table.*

---

Replacing Tooth on Vulcanite Plate. Drill a hole through the vulcanite where the pins were located with a number eight bur. Ream the hole on the palatal surface with a larger bur to provide a clincher space. Set the tooth in proper place and cover it with Mouldine—modeling compound will answer—to hold it in place. Then make a funnel-shaped duct on the palatal side of the mould to pour the metal into. Melt Melott's metal or Watt's metal in a spoon and pour into the hole. When cool remove the

Mouldine and finish to proper form.—W. S. Walters, in *Summary*.

---

MALFORMED TEETH. Dr. G. B. McClintock, in the *Journal A. M. A.* for Aug. 9, says he has noticed during several years of examination of radiograms of degenerate epileptics and others mentally weak, that the teeth are malformed or lacking in complete development both of the crowns and roots. If the roots are full formed they are short or blunted to a marked extent. Has anyone noticed this; if so, what is the explanation?

---

PULP-CANAL TREATMENT. It has been said sometimes that it takes a long time to gain access to the canal, and that one might work with sulfuric acid for several hours before access is gained to the canal. It reminds me of a story Dr. McCollum told me of a letter that he received from a man, who wrote very indignantly and said, "I am absolutely disgusted with your treatment of sulfuric acid or the handling of sulfuric acid; there is nothing to it at all. I used sulfuric acid in a tooth for six months and finally the whole darn'd crown fell off and it hadn't opened up a single root-canal."

Another good friend of mine I thought got off a very good one when he says, "But I don't notice you say anything about the rubber dam." He said, "One of my professors in college said one thing that I will always remember that man for: 'If you can't,' he said, 'and by that we mean, if you have employed all the ingenuity you can muster, if you can't get a rubber dam on, the tooth isn't worth a damn.' " And you know, the more I think of that, the more I believe that that fellow was right, that if, after we have employed all the ingenuity that we ourselves can originate, and that we can borrow, beg or steal, and that is where we get the most of it, from the other man, for, after all, there is very little original in this world you know, if you can not get the rubber dam on that tooth, there are mighty few of them I would try to save. How can we overcome that? In the first place, the case of a decayed tooth that has been cut off close to the gum line; there are clamps which go down underneath the very margin of the gums. These in many cases will aid one in getting the rubber dam on the tooth. For the tooth that is badly broken down there is another method. You can place on that tooth a copper band and pack amalgam down between the band and the root and make the band a little larger than the tooth, and force it down with considerable pressure to tighten the band in all directions. But remember that whenever it is possible the clamp should be applied to the tooth distal to the one to be operated upon. Another way is to take a copper band and by putting some cement around the tooth, and cementing it on. An aluminum band may also be put around the tooth; and by means of gutta-percha, packed down in between the band and the tooth, it is stretched tightly against the root. I realize that a great many men are using arsenic, but I still maintain that I am not in on the secret. I have not had it explained to me this morning how it is safe, how we control the action of the arsenic. Pressure anesthesia following arsenic would force the arsenic through the end of the root, and that is something that I would not care to do. I would not feel justified in explaining to the patient the chances I was taking and ask them to submit to them; and anyway, pressure anesthesia is not indicated in those cases. I did not say anything about pressure anesthesia, but I say I have observed some cases where pressure anesthesia was used and the next day when we radiographed the teeth, we found beautiful areas around the end of those roots. Now, something happened there that we had not planned on. I have opened many of those cases and found the whole pulp packed down in the apical portion of that root, and I am afraid of pressure anesthesia almost as much as I am of arsenic.—Dr. E. Best, in *Pacific Gazette*.

